# Epigenetic Induction of EGR-1 Expression by the Amyloid Precursor Protein during Exposure to Novelty

**DOI:** 10.1371/journal.pone.0074305

**Published:** 2013-09-16

**Authors:** Aurélie Hendrickx, Nathalie Pierrot, Bernadette Tasiaux, Olivier Schakman, Jean-Pierre Brion, Pascal Kienlen-Campard, Charles De Smet, Jean-Noël Octave

**Affiliations:** 1 Institute of Neuroscience, Université catholique de Louvain, Brussels, Belgium; 2 Laboratory of Histology and Neuropathology, Université libre de Bruxelles, Brussels, Belgium; 3 de Duve Institute, Université catholique de Louvain, Brussels, Belgium; Nathan Kline Institute and New York University School of Medicine, United States of America

## Abstract

Following transcriptome comparison of primary cultures isolated from brain of mice expressing or not the amyloid precursor protein APP, we found transcription of the EGR-1 gene to be regulated by APP. In primary cultures of cortical neurons, APP significantly down regulated EGR-1 expression at both mRNA and protein levels in a γ-secretase independent manner. The intracellular domain of APP did not interact with EGR-1 gene promoter, but enrichment of acetylated histone H4 at the EGR-1 promoter region was measured in APP-/- neurons, as well as in brain of APP-/- mice, in which increase in EGR-1 expression was also measured. These results argue for an important function of APP in the epigenetic regulation of EGR-1 gene transcription both in vitro and in vivo. In APP-/- mice, constitutive overexpression of EGR-1 in brain impaired epigenetic induction of this early transcriptional regulator during exposure to novelty. Altogether, these results indicate an important function of APP in the epigenetic regulation of the transcription of EGR-1, known to be important for memory formation.

## Introduction

Among the various functions that have been attributed to APP, regulation of gene expression has been widely investigated due to similarity between Notch and APP processing. The γ-secretase cleavage of APP and Notch releases the APP intracellular domain (AICD) and the Notch intracellular domain (NICD), respectively. Processing of Notch is initiated by binding of the Delta ligand on one cell to the Notch receptor on another cell resulting in two proteolytic cleavages of the receptor. The α-secretase cleavage generates a substrate for cleavage by the γ-secretase complex. This proteolytic processing mediates release of the NICD, which enters the nucleus to regulate transcription [1]. APP has all the characteristics of a type1 transmembrane receptor, and interacts with many proteins via its extracellular domain [2]. APP and Notch are processed by the same secretase activities and APP has been suggested to control transcription of several genes [3-8]. To study regulation of gene transcription by APP, transcriptomes of primary cultures isolated from brain of APP+/+ and APP-/- mice from the same genetic background were compared using the affymetrix microarray technology, and we identified EGR-1 as a possible target gene controlled by APP.

EGR-1 (also called Zif 268, Krox24, NGFI-A) is a an early growth response gene, member of the zinc finger family of transcription factors that displays fos-like induction kinetics in many cells including neurons. EGR-1 regulates transcription of late response genes important for the synaptic plasticity processes, especially the maintenance of long-term potentiation [9]. In mouse brain, EGR-1 is constitutively expressed in the cortex but is rapidly induced in the prefrontal cortex and hippocampus after several stimuli as exposure to novelty, fear conditioning or recognition task[10-12]. .In addition, EGR-1 up regulates presenilin-2 gene expression in neuronal cells [13], and consequently the γ-secretase cleavage of APP. Moreover, EGR-1 is up regulated in brain of patients with Alzheimer disease (AD), and overexpression of EGR-1 controls both phosphorylation and dephosphorylation of tau, by activating CDK5 and inactivating PP1, leading to tau hyperphosphorylation and destabilized microtubules [14]. For all these reasons, we were interested to study how APP is involved in the regulation of EGR-1 gene transcription.

Our results indicate that both in cultured neurons and in vivo, APP affects EGR-1 expression by modulating enrichment of acetylated histone H4 on the EGR-1 gene promoter, and therefore participates to the epigenetic regulation of EGR-1 expression. The constitutive overexpression of EGR-1 in APP -/- mice impairs induction of this early transcriptional regulator during exposure to novelty.

## Materials and Methods

### Cell culture and pharmacological treatments

Primary cultures of cortical astrocytes were prepared from newborn p-0/p-1 mice pups as previously described [15]. APP +/+ and APP -/- pups from the same littermate were genotyped and sexed. Briefly, cortices were isolated and dissociated in Dulbecco’s modified Eagle medium DMEM (glutaMAX) supplemented with 10% (FBS), proline (50 mg/ml), penicillin-streptomycin (50 mg/ml), and fungizone (2.5 mg/ml). After centrifugation, cells were seeded into culture flasks grown at 37°C in a 5% CO2 atmosphere during 7 days. At this time point, culture flasks were vigorously shaken to eliminate cell debris, microglia, and oligodendrocytes. Three days later, cells were plated at 10^4^ cells/cm^2^ in culture dishes pre-treated with 10mg/ml poly-L-lysine in PBS.

Primary cultures of cortical neurons were performed on p-0/p-1 newborn pups from APP +/+ and APP -/- mice. Briefly, cortices were digested in trypsin/DNAse medium (PBS containing 10 mg/ml trypsin; 1mg/ml DNAse; 6mM NaOH). Then, they were dissociated with a glass pipette in Neurobasal medium supplemented with 2% v/v B27 medium, 0.5% L-glutamine, 0.1% Pen-strep. All reagents were obtained from Invitrogen. Cortical neurons were grown on 6 or 12 wells culture dishes pre-coated with 10 µg/ml poly L-Lysine, at a density of 4x10^5^cells/cm^2^ and cultured during 5 days in Neurobasal medium. On day 4, neurons were treated 24h with the HDACs inhibitor, trichostatin A at final concentration of 25ng/ml (Sigma) or vehicles. Neurons at DIV5 were treated during 8h with DAPT (250 nM) a functional γ-secretase inhibitor.

### Animals and tissue samples

The APP+/+ and APP -/- mice used in this study were C57Bl/6J obtained from The Jackson laboratory and backcrossed for more than 5 generations in the CD1 genetic background. The open-field test was used to assess the exposure of mice to a new environment. Mice had access to food and water ad libidum, and they were moved to the experimental platform animal house one week before the open field. Mice were exposed to open field area during 15 min and then sacrificed 30 min after this exposure to enable the expression of EGR-1. All manipulations on mice have been approved by the local ethics committee of the catholic University of Louvain and follow the European legislation.

Postmortem human brains (n=6) were collected with the approval of the Ethical Committee at the Medical School of the Free University of Brussels and have been described previously [16]. One hemisphere was frozen in liquid nitrogen and stored at -80°C for biochemical analysis. The other hemisphere was fixed by immersion in 10% formalin (v/v) for neuropathological analysis.

### RNA extraction and quantitative real time PCR

Total RNA was purified using Trizol method (Tripure, Roche). Reverse transcription (RT) and quantitative (q) real time PCR (q-RT PCR) were respectively performed with the iScript cDNA synthesis Kit and the iQ SYBR Green supermix using a iCycler MyIQ2 multicolor Real-Time PCR detection system (Biorad). The relative amplification of cDNA fragments was calculated by the 2-ΔΔCt method. q-RT PCR primer sequences used were as follows: EGR-1 forward : TCCTCTCCATCACATGCCTG, EGR-1 reverse: CACTCTGACACATGCTCCAG, GAPDH forward: ACCCAGAAGACTGTGGATGG, GAPDH reverse: ACACATTGGGGGTAGGAACA.

### Protein analysis

Cells or powdered frozen tissues were lysed in a Laemmli buffer (125mM Tris pH 6.8, 20% glycerol, 4% SDS) with complete protease inhibitor cocktail (Roche). For nuclear extraction, material was lysed in 0.25 M sucrose buffer (sucrose 0.25 M, Tris 50mM, EDTA 1mM, imidazole 3mM, pH 7.0 + proteases inhibitor cocktail), and centrifuged 10 min at 250g. Nuclear fraction was resuspended in Laemmli buffer. Western blotting was performed on 10 µg of protein lysates. All samples were sonicated before protein assay (BCA Pierce, Thermoscientific), incubated during 5 min at 95°C in Laemmli containing DTT and staining blue (Nupage blue, Invitrogen), loaded onto 4–12% NupageTM bis-Tris gel electrophoresis (Invitrogen), and then transferred to nitrocellulose membranes (Amersham Biosciences). Membranes were incubated overnight at 4°C on a wheel with the primary antibodies; anti-APP C-ter 1:3500 (kind gift of N. Sergeant, INSERM U422, Lille, France), anti-EGR-1 1:1000 (sc-110 Santa Cruz), anti-tubulin 1:6500 (Sigma). Washes with PBS-Tween (0.005%) were followed by incubation with secondary antibody (1:10 000 anti-mouse or anti-rabbit IgG) (GE Healthcare) coupled to horseradish peroxidase and revealed by ECL. For quantification, the membranes were stripped and reincubated with an anti-tubulin. Immunoreactive bands were quantified with an electrophoresis Gel Doc 2000 imaging system coupled to a Quantity one™ software (Bio-Rad).

### Aβ measurement by ECLIA

We measured the concentration of mouse Aβ 40 in culture medium from neurons treated or not with DAPT. Samples were centrifuged at 12000g during 3 min at 4°C, and Aβ 40 was quantified in 25 µl of culture medium by multiplex Electro-Chemiluminescence ImmunoAssay (ECLIA) (Meso scale Discovery), analyzed with the Meso Scale Discovery (MSD) SECTOR™ Imager 2400 according to manufacturer’s instructions.

### Chromatin immunoprecipitation

Chromatin immunoprecipitation was performed on cortical neurons (day 5) or mice cortices using the EZ-ChIP Assay kit (Millipore) according to manufacturer instructions. Cortices were powdered in liquid nitrogen and DNA-protein interactions were directly cross-linked by the addition of a 37% v/v of formaldehyde 37% (Sigma) into PBS 1X containing protease inhibitor cocktail during 20 minutes. Cells seeded in 6 wells plaques were pelleted and resuspended in Dulbecco modified Eagle’s medium (DMEM) before crosslink with formaldehyde 37% during 10 minutes. The crosslinking reaction was stopped by adding glycine. After several washes with cold PBS and resuspension in SDS lysis buffer containing inhibitors of proteases, samples were sonicated in order to shear the cross linked chromatin. Conditions of sonication were experimentally adapted given the cell type, culture conditions, and equipment. Cells and brains were sonicated in an ice bath at high power and a series of 30 cycles of 30 s for neurons and 25 cycles for cortices, and were performed with a sonicator “Bioruptor® UCD-20’” (Diagenode). The samples were kept on ice during 30 s between two pulses. Verification of sheared DNA on 1.2% agarose gel was realized after incubation of an aliquot with RNAse A at 37°C during 30 min and with proteinase K at 65°C during two hours. We obtained a sheared DNA with an average size of fragments between 400 and 200 pb. An aliquot of precleared DNA was collected as the input. The samples were then pre-cleared with ChIP blocked protein G agarose and incubated overnight at 4°C with the antibodies of interests (5μg anti-AcH3 (07-690 Millipore); 5μg anti-AcH4 (06-599 Millipore); 10µl anti-APP Cter serum (kind gift of N.Sergeant) or 10µg of the commercially available anti-APP Cter antibody (A8717 Sigma). After immunoprecipitation, the DNA–histone complex was collected with 40 µl of ChIP blocked protein G agarose beads for 1h. The beads were sequentially washed once with low salt, high salt, and LiCl and washed twice with 10 mM Tris (pH 8)/1 mM EDTA buffers. DNA was finally collected with spin filters, and the immunoprecipited DNA was analyzed by qPCR with primers designed to amplified short regions of the promoters of genes of interest.

qPCR primers were as follows: EGR-1 forward: GTGCCCACCACTCTTGGAT, EGR-1 reverse: CGAATCGGCCTCTATTTCAA, GAPDH forward: AGAGAGGGAGGAGGGGAAATG, GAPDH Reverse: AACAGGGAGGAGCAGAGAGCAC. The quantification method used is based on the ratio between immunoprecipitated DNA and input DNA. The method was validated by the amplification of the well characterized GAPDH gene.

### Statistical analysis

All results are expressed as mean ± standard error (SE) values. Statistical significance was determined by one-way or two-way analysis of variance (ANOVA) followed by Bonferroni’s multiple comparisons test for multi-group comparison and the student’s t-test for two-group comparisons.

## Results

### 1. Transcriptome comparison of primary cultures isolated from brain of APP+/+ and APP-/- mice

High quality RNA was prepared from expanded primary cultures of astrocytes from APP+/+ and APP-/- mice from the same genetic background, and utilized in the Affymetrix platform for transcriptome comparison. As a positive control, APP appeared more than strongly expressed in APP+/+ astrocytes. In addition to APP, a list of genes with up regulated transcription in APP+/+ astrocytes is presented in table 1. Transcription of several genes was also up regulated in APP-/- astrocytes (table 1). Although up regulation of EGR-1 gene transcription in APP-/- cells was not impressive, this gene encodes an early transcription factor involved in memory formation, and we further investigated the regulation of EGR-1 expression by APP in primary cultures of cortical neurons.

**Table 1:** Microarray data.

	**Fold Changes**	
**Gene symbol**	**APP +/+**	**APP -/-**
Slc46a1	22,0	
Kcnf1	15,3	
Myb	14,3	
2210409E12Rik	13,6	
Ptger1	12,8	
Tcta	12,5	
Ypel1	12,1	
Dmkn	10,6	
Colec11	10,1	
BC003883	9,9	
4930578N16Rik	8,7	
1700026D08Rik	8,4	
4930579K19Rik	7,8	
Dnaic2	7,7	
Scpep1	7,5	
Lsm14a	7,2	
Mgp	7,0	
Adi1	6,9	
Rbm47	6,4	
Prdm16	6,3	
Rnf39	6,2	
C81615	6,1	
Trp73	6,1	
629242 /// BC005512 /// F630007L15Rik		16,7
Cuedc1		9,3
Tubb2c /// Tubb2c-ps2		6
Cenpe		5,4
Fam115a		5,3
Slc15a2		5,2
Ranbp2		5,1
Aldh1a7		4,9
Malat1		4,8
Slc15a2		4,6
Neo1		4,6
Cdc40		4,6
Tubb2a-ps2		4,3
Klhdc7a		4,3
Zfp455		4,2
AU015680		4,2
Eif5b		4,2
Pcdhb21		4,2
Vps37a		4,2
Cpox		4,2
2210018M11Rik		4,1

mRNA from APP+/+ and APP-/- astrocytes was utilized in the Affymetrix platform for transcriptome comparison. Genes are notified by gene symbol and increase in gene transcription is indicated as fold changes in APP+/+ or APP-/- astrocytes.

### 2. APP down regulates neuronal EGR-1 expression both at the mRNA and protein levels

**Figure 1:**
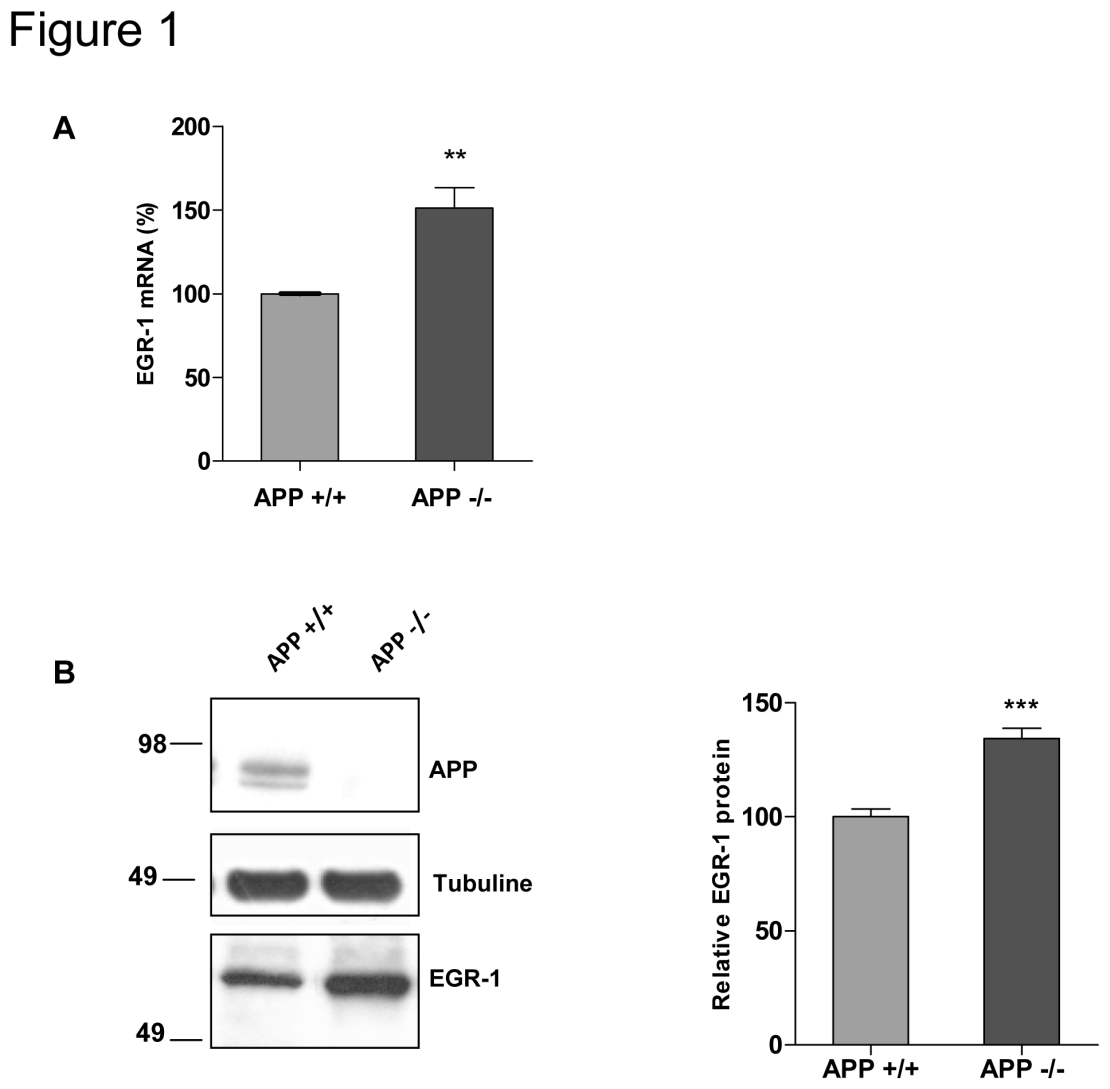
APP regulates EGR-1 expression at both mRNA and protein level. A) EGR-1 mRNA levels were quantified by q-RT PCR in APP+/+ and APP-/- neurons DIV5 (n=9). Values were normalized to the GAPDH mRNA, and expressed as percentage of APP+/+. Student’s t-test : **p<0.005. B) EGR-1 protein was detected by Western blots of total and nuclear extracts obtained from primary culture of neurons DIV5 (n=3). APP was detected in APP+/+ neurons with an anti-APP C-terminal antibody, EGR-1 was detected with sc-110 antibody and anti-tubulin antibody was utilized for loading control. ***p<0.001. Data are expressed as mean ± SEM.

To confirm data obtained from the comparison of transcriptomes using Affymetrix technology, primary cultures of cortical neurons from APP+/+ and APP-/- mice from the same genetic background were analyzed using q-RT PCR and western blotting. Results presented in Figure 1A indicate that EGR-1 mRNA was significantly more abundant in APP-/- than in APP+/+ neurons. At the protein level, a significant decrease in EGR-1 was also quantified in APP+/+ as compared to APP-/- neurons (Figure 1B). From these results, we conclude that APP down regulates neuronal EGR-1 expression both at the mRNA and protein levels.

### 3 APP-mediated regulation of EGR-1 expression is independent of the γ-secretase cleavage

**Figure 2:**
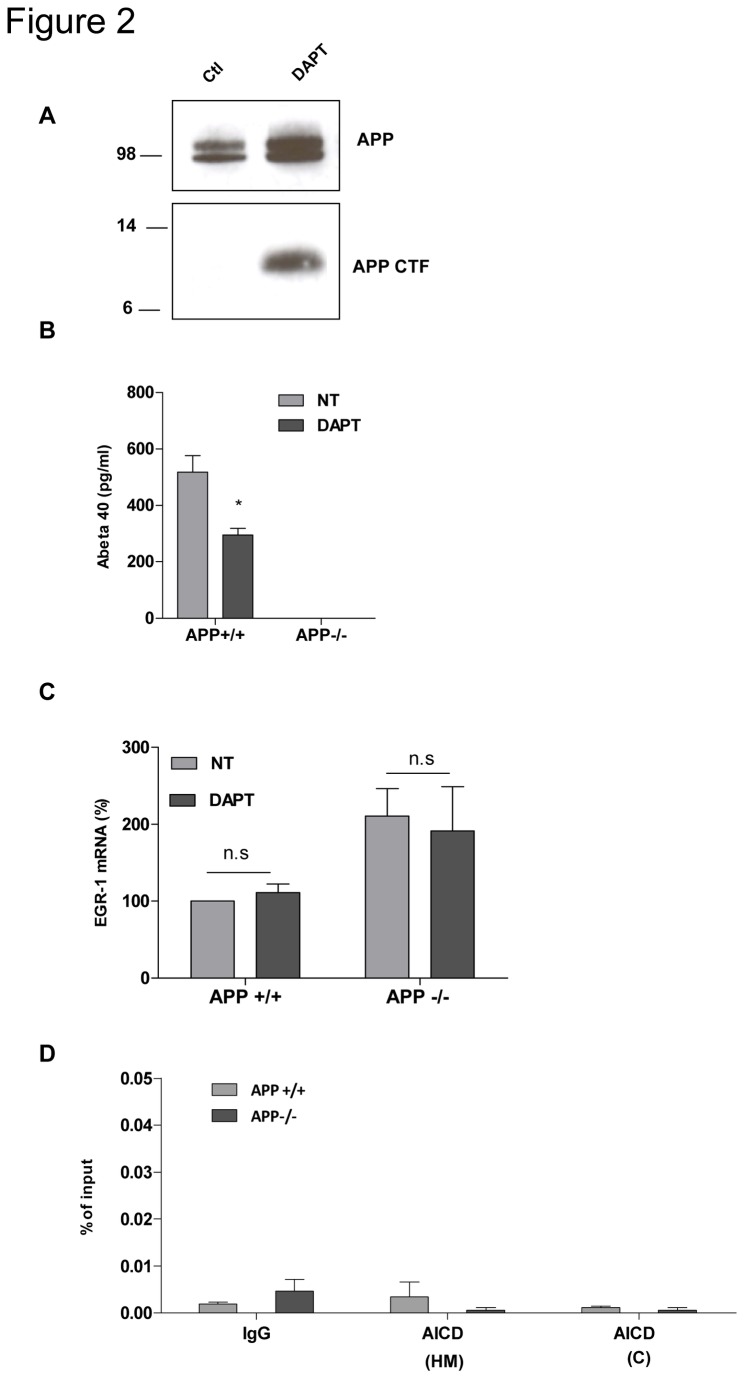
Pharmacological inhibition of γ-secretase does not affect APP-mediated control of EGR-1 expression. A) Western blot analysis of cellular extracts from control neurons (Ctl) or neurons treated for 8h with 250nM DAPT (DAPT). APP and APP CTF were detected by with an antibody raised against the C-terminal domain of APP. B) Aβ was quantified in the culture medium of APP+/+ or APP-/- neurons treated (DAPT) or not (NT) for 8h with 250nM DAPT. C) EGR-1 mRNA levels were quantified by q-RT PCR on APP +/+ and APP-/- neurons DIV5 treated (DAPT) or not (NT) for 8h with 250nM DAPT (n=3). Values were normalized to the GAPDH mRNA, and expressed as percentage of APP+/+. Results are expressed as mean ± SEM. D) Chromatin immunoprecipitation of EGR-1 promoter was performed on chromatin isolated from APP+/+ and APP-/- neurons DIV5 (n=3) using normal mouse IgG as negative control (IgG) and two different anti-C-terminal APP antibodies: anti-AICD homemade (HM) or commercial (C). Immunoprecipitated DNA was quantified by qPCR, and normalized to the input.

Since the intracellular domain of APP (AICD) is a transcriptional regulator, we next studied whether it could account for APP-dependent repression of EGR-1 gene transcription. Rescue of APP by adenoviral expression of APP or APP deleted from AICD in APP-/- neurons did not allow us to conclude. Unfortunately, up regulation of EGR-1 expression in these experimental conditions was proportional to the adenoviral load rather than to the protein expressed (data not shown). Therefore, we decided to inhibit AICD release by the γ-secretase inhibitor, DAPT [17], which induced a very strong accumulation of APP C-terminal fragments in APP+/+ neurons (Figure 2A), resulting from the inhibition of the γ-cleavage of APP. DAPT did not affect APP-mediated down regulation of EGR-1 mRNA levels (Figure 2C). In addition, the γ-secretase inhibitor decreased by 50% Aβ secretion (Figure 2B), without affecting EGR-1 mRNA levels in APP+/+ neurons. We then performed chromatin immunoprecipitation assays using antibodies raised against the C-terminal domain of APP (AICD). These antibodies failed to enrich EGR-1 promoter sequences in both APP+/+ and APP-/- neurons (Figure 2D). Altogether, these results rule out that APP-mediated decrease in EGR-1 mRNA levels is γ-secretase dependent, and indicate that EGR-1 expression is not regulated by extracellular Aβ. In addition, AICD does not interact with the EGR-1 promoter.

### 4. Acetylated histone H4 is enriched on the EGR-1 gene promoter in APP-/- neurons

**Figure 3:**
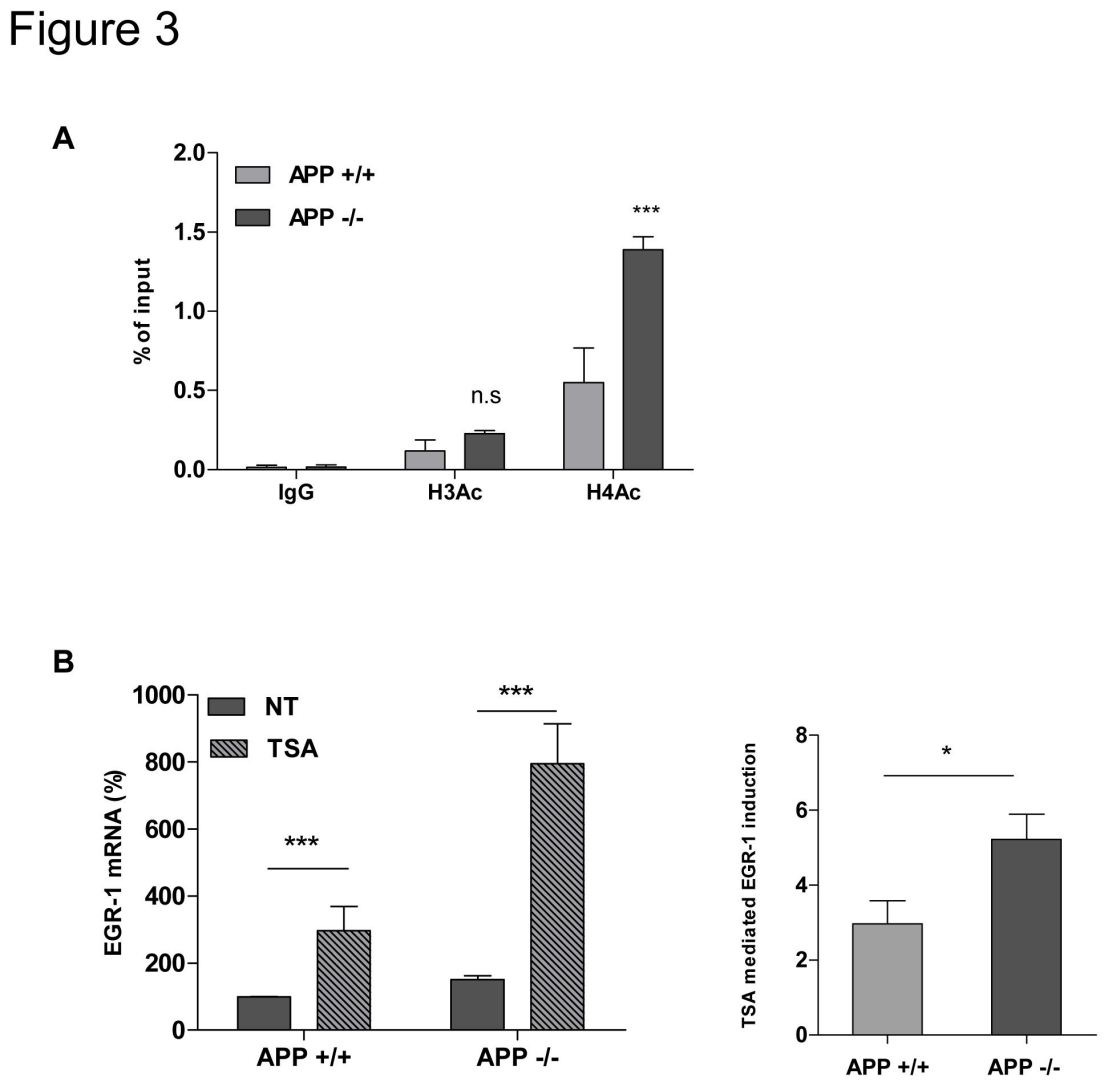
Epigenetic regulation of EGR-1 gene expression by APP. **A**. Fragmented chromatin obtain from APP+/+ and APP-/- neurons DIV5 (n=3) was immunoprecipitated with antibody recognizing normal mouse IgG as negative control (IgG), anti-acetylated histone H3 (H3Ac) or anti-acetylated histone H4 (H4Ac). Quantification was realized by real-time qPCR performed on immunoprecipitated DNA with primers designed on EGR-1 promoter, and normalized to the input. n.s. non-significant. ***p<0.001. **B**. EGR-1 mRNA levels were quantified in APP+/+ and APP-/- neurons DIV5 treated (TSA) or not (NT) for 24 h with 25ng/ml TSA (n=6). Values were normalized to the GAPDH mRNA, and expressed as percentage of APP+/+. Two way Anova. **p<0.005, ***p<0.001. Relative induction of Egr-1 after exposure to TSA after normalization. Student t test. *p<0.05.

Epigenetic regulation of EGR-1 gene transcription has been previously demonstrated, in particular by modulation of enrichment of acetylated histones on the EGR-1 promoter by HDAC 2 activity [18]. Chromatin immunoprecipitation assays using anti-acetylated histone antibodies showed that acetylated histones H3 and H4 were enriched at the EGR-1 gene promoter, but acetylated histone H4 was more enriched in APP-/- neurons (Figure 3A). Consistent with the notion that acetylation of histones opens chromatin and improves gene transcription, more acetylated histone H4 at the EGR-1 promoter could improve EGR-1 expression in APP -/- neurons. These results demonstrate that APP significantly decreases enrichment of acetylated histone H4 at the promoter of EGR-1 gene, leading to reduced EGR-1 gene transcription.

A difference in histone acetylation at the promoter of EGR-1 gene should influence the effect of an HDAC inhibitor on EGR-1 gene transcription in APP+/+ and APP-/- neurons. To test this hypothesis, the effect of trichostatin A (TSA) on EGR-1 gene transcription was investigated in both APP+/+ and APP-/- neurons. Results presented in Figure 3B indicate that TSA was able to induce EGR-1 transcription in both APP+/+ and APP-/- neurons, although induction was significantly higher in APP-/- neurons, in agreement with higher H4 acetylation at the EGR-1 promoter in these cells.

### 5. In vivo regulation of EGR-1 expression by APP

**Figure 4:**
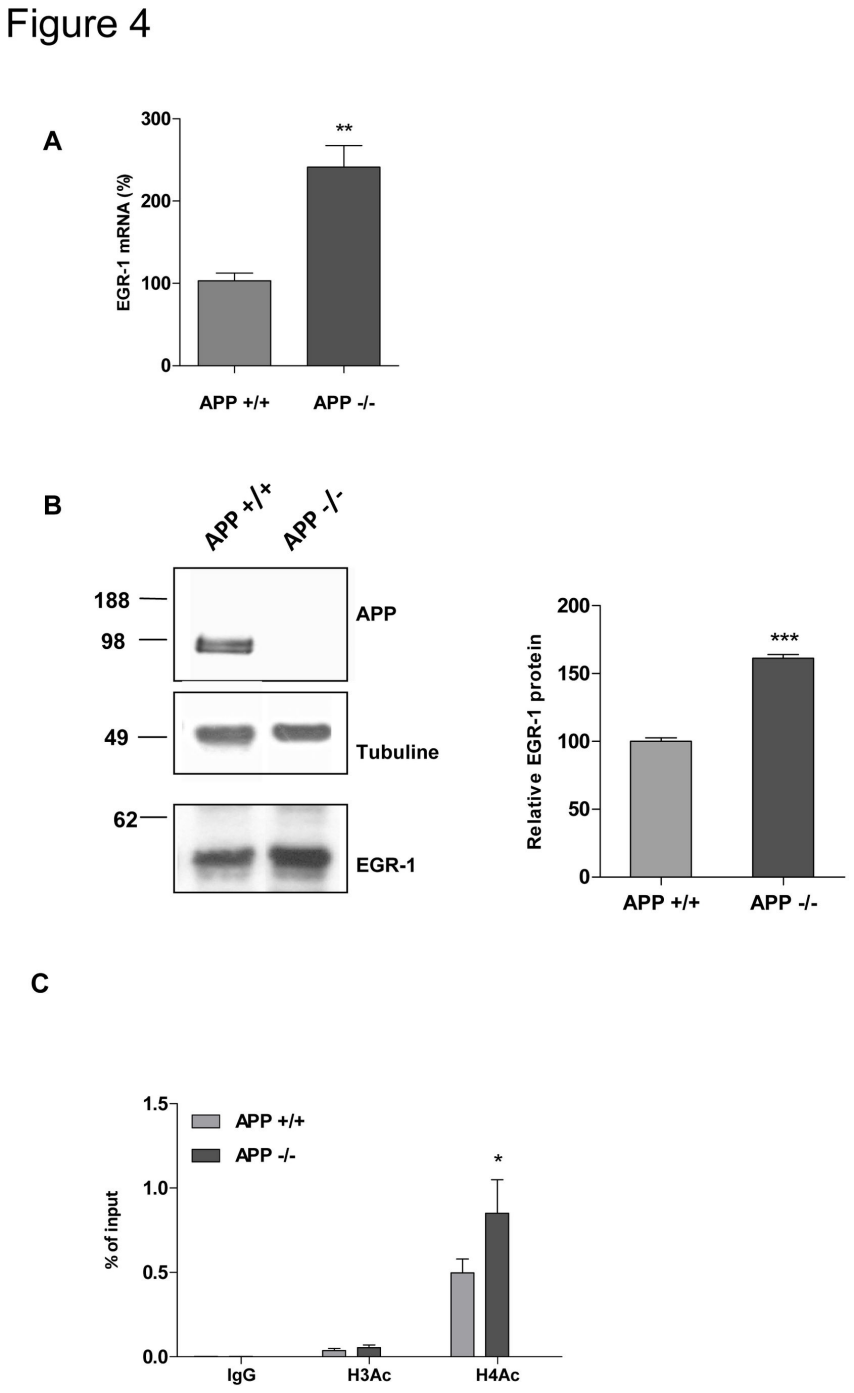
APP controls EGR-1 expression *in vivo*. **A**) EGR-1mRNA levels were quantified by q-RT PCR in mouse cortex (n=9). Values were normalized to the GAPDH mRNA, and expressed as percentage of the APP+/+ tissue. Student’s t-test: ***p < 0.001. **B**) EGR-1 protein expression was detected by Western blot analysis of extracts obtained from cortex of APP +/+ and APP -/- mice, 5 months of age (n=5). Typical blot is shown with anti-tubulin utilized as a loading control. EGR-1/tubulin ratio were quantified, and expressed as percentage of the APP+/+ mice. Student’s t-test : ***p<0.001. Data are expressed as mean ± SEM.C) Chromatin immunoprecipitations of EGR-1 promoter were performed on chromatin isolated from mice cerebral cortex (n=7) with normal IgG as negative control, anti-acetylated H3 antibody, or anti-acetylated H4 antibody. Immunoprecipitated DNA was quantified by qPCR and normalized to the input. Two way Anova, n.s. non-significant, *p<0.05.

To further investigate whether APP-mediated regulation of EGR-1 expression in cultured neurons was relevant in vivo, cerebral cortices of APP+/+ and APP-/- mice were analyzed in q-RT PCR and western-blotting. Results presented in Figure 4A clearly demonstrate up regulation of EGR-1 mRNA levels in brain of APP-/-, which was very similar to that observed in cultured neurons. At the protein level, a significant increase in EGR-1 was also measured in brain of APP-/- mice (Figure 4B). To investigate whether APP-mediated down regulation of EGR-1 in vivo could occur at the epigenetic level, chromatin immunoprecipitation assays were performed on brain samples from APP+/+ and APP-/- mice. Results presented in Figure 4C indicate that acetylated histone H4 was more enriched at the EGR-1 gene promoter in APP-/- than in APP+/+ brain, confirming results obtained in cultured neurons. Altogether, these results allow us to conclude that APP participates in the epigenetic regulation of EGR-1 gene transcription both in vitro an in vivo.

### 6. Physiological relevance of APP-mediated regulation of EGR-1 expression

**Figure 5:**
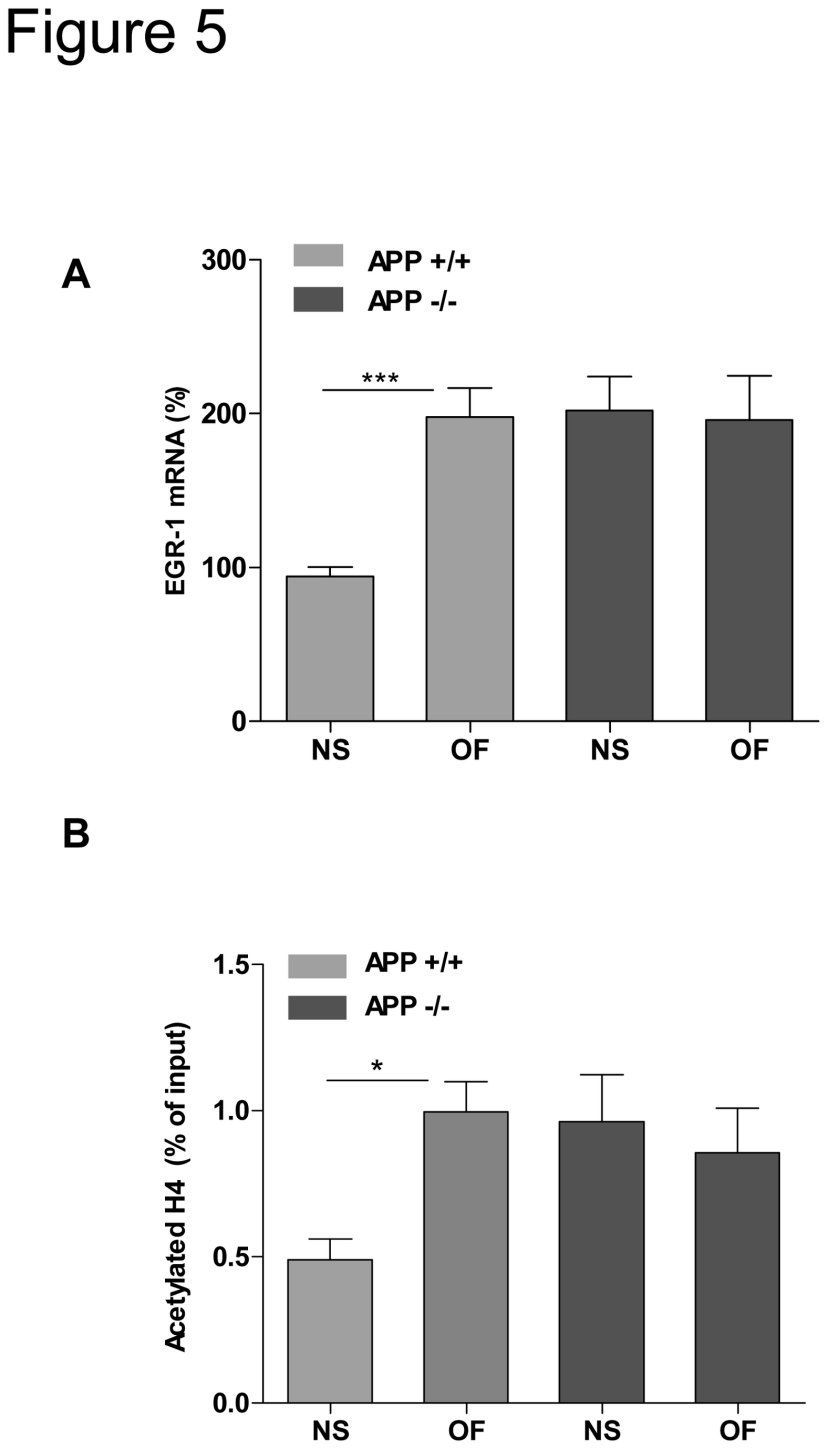
APP is needed to induce EGR-1 by novelty. **A**) EGR-1mRNA levels were quantified by q-RT PCR in cortex of APP+/+ and APP-/- mouse exposed (OF) or not (not stimulated: NS) to the open field during 15 min (n=10). Values were normalized to the GAPDH mRNA, and expressed as percentage of the APP+/+ NS. One way Anova (***p<0.001) **B**) Chromatin immunoprecipitations of EGR-1 promoter were performed on chromatin isolated from cerebral cortex APP +/+ and APP -/- mice exposed or not to novelty (n=7 per group) with normal IgG as negative control and anti-acetylated H4 antibody. Immunoprecipitated DNA was quantified by qPCR and normalized to the input. Two way Anova, *p<0.05.

Several studies indicate that immediate early genes, and in particular EGR-1, play an important role in learning and memory processes, and that EGR-1 expression is rapidly up regulated during exposure to novelty [19]. Therefore, APP+/+ and APP-/- mice were exposed or not to an open field during 15 minutes and EGR-1mRNA levels were thereafter quantified in the cerebral cortex. Results presented in Figure 5A indicate a significant induction of EGR-1 gene transcription in APP+/+ but not in APP-/- mice, suggesting that constitutive high expression of EGR-1 in APP -/- mice could impair induction of this early transcriptional regulator during exploration behavior. Moreover, exposure to novelty induced significant enrichment of histone H4 acetylation on EGR-1 promoter region only in APP+/+ mice (Figure 5B). All together, these results indicate that APP is needed to induce epigenetic expression of EGR-1 during exposure to novelty.

### 7. Up regulation of EGR-1 in brain of patients with Alzheimer disease

**Figure 6:**
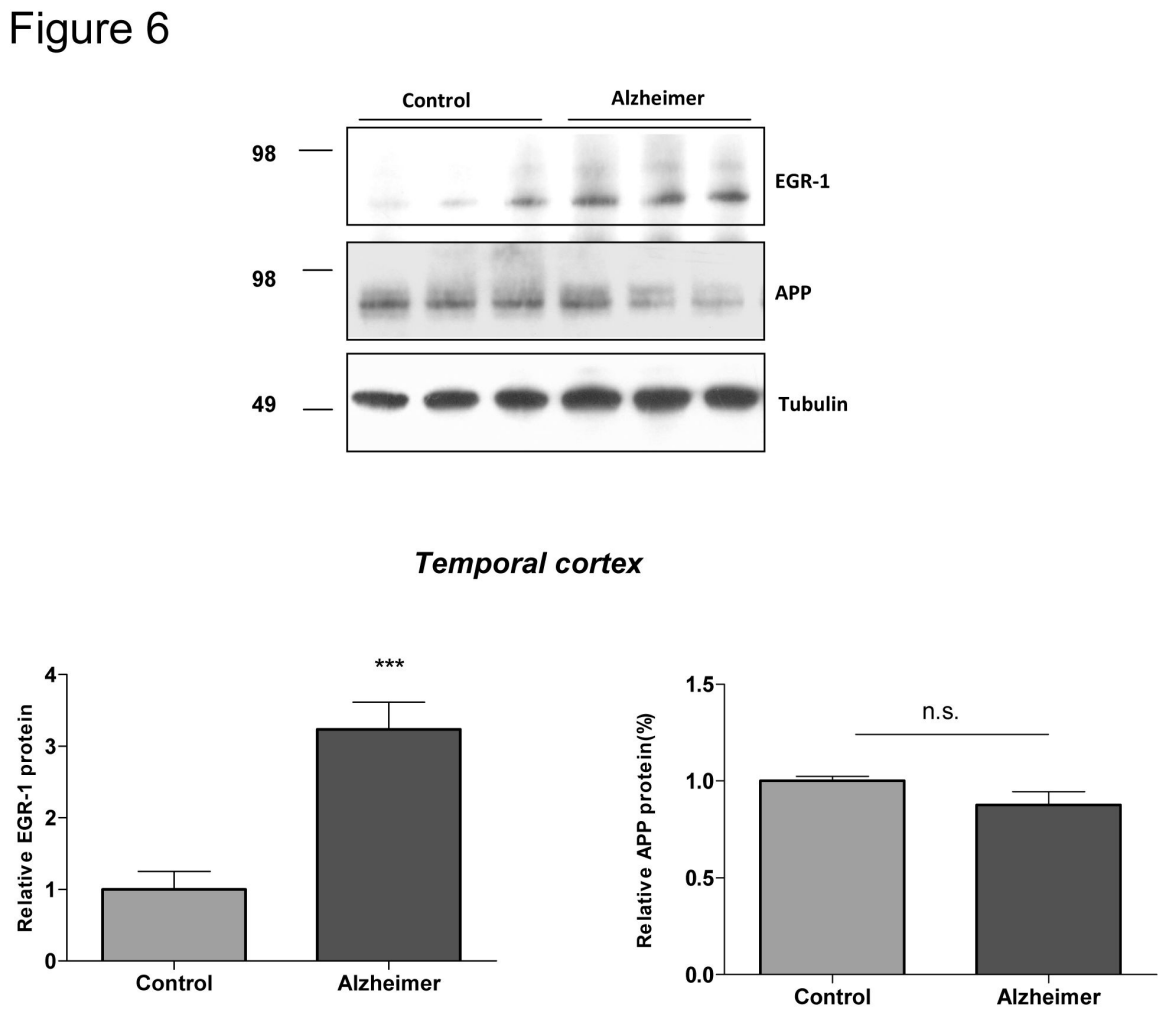
EGR-1 is up-regulated in Alzheimer’s disease. Egr-1 and APP protein levels were measured in AD (n=3) versus control (n=3) brains by Western blotting. Analyses were performed on total lysates of temporal cortex. Egr-1 was detected with sc-110 antibody, APP with anti-APP C terminal (HM) and anti-tubulin was utilized as a loading control.

EGR-1 was previously described to be up regulated in brain of AD patients [14,20,21]. Since our results indicate that APP epigenetically down regulates EGR-1 gene transcription, we wondered whether up regulation of EGR-1 in AD could be related to down regulation of APP. Control and AD brains were analyzed in western blotting using anti-EGR-1 and anti-APP antibodies. Results presented in Figure 6 confirm up regulation of EGR-1 in AD brains. However, a concomitant significant decrease in APP was not observed. These results suggest that up regulation of EGR-1 in AD is APP independent, or that a particular function of APP is lost in AD, resulting in up regulation of EGR-1 expression similar to that found in APP knockout mice.

## Discussion

Our findings demonstrate that both in cultured neurons and in vivo, APP is able to significantly down regulate EGR-1 expression. In both non amyloidogenic and amyloidogenic catabolic pathways, APP is cleaved by the γ-secretase activity to release the APP intracellular domain (AICD), which is able to regulate transcription of several genes, including APP itself, the GSK3β, and the Aβ-degrading enzyme neprilysin [3,4,22,23,6,24,25]. It has been however established that nuclear location of AICD and consequent transcriptional regulation is dependent on APP processing through the amyloidogenic pathway [25,26,27]. Although regulation of gene transcription by AICD has been a matter of debate [28,29], probably reflecting the cell specificity of AICD regulation [30,31,32], it has been demonstrated by chromatin immunoprecipitation studies that AICD does interact with promoter regions of putatively regulated genes [24,25]. Here, we have demonstrated that the cleavage of APP by the γ-secretase activity to release AICD is not necessary for APP-mediated regulation of EGR-1 expression, and that AICD does not interact with the EGR-1 promoter in chromatin immunoprecipitation studies. APP mediates epigenetic control of a number of genes, including the AQP1 gene [8], at least in part through displacement of histone deacetylases (HDAC) [24]. In agreement with these data, we demonstrate here an enrichment of acetylated H4 on the EGR-1 promoter, both in APP-/- cultured neurons and in APP-/- mouse brain, arguing for the epigenetic control of EGR-1 expression by APP. Acetylation of H4, in particular at position K12, has been previously reported to increase transcription of memory related genes and mice unable to induce acetylation of H4K12 show learning and memory impairments [33]. Our preliminary results indicate that acetylated H4K12 is enriched on the EGR-1 promoter in absence of APP. Acetylation of H4 at position K12 is highly regulated by HDAC2 activity [18]. Whether APP affects expression or cellular localization of HDAC2 deserves further investigations.

In some cases, other APP metabolites, and in particular soluble APPs, are also able to control gene expression [34]. Recently, secreted β- and α-APP were reported to induce axon outgrowth in vitro through EGR-1 signaling [35]. In this study, bacterial recombinant sAPPα or sAPPβ added to primary neuronal cultures were able to stimulate EGR-1 expression. In our study, the culture medium recovered from APP+/+ neurons was not able to modify EGR-1 expression in APP -/- neurons (data not shown).

Induction of EGR-1 is required in the hippocampus and in the cortex for the formation of memory and late long term potentiation [9]. In mouse brain, EGR-1 is induced following 15 minutes exposure to novelty in an open field [36]. Accordingly, we were able to induce EGR-1 expression in brain of APP+/+ mice during a 15 min exposure to novelty, and this induction was concomitant with enrichment of acetylated H4 at the EGR-1 promoter. In marked contrast with APP+/+ mice, APP-/- mice were unable to induce EGR-1 expression during exposure to novelty, readily explained by the high basal level of EGR1 expression in these mice. APP could therefore play a major role as an epigenetic regulator of transcription of genes involved in memory formation. EGR-1 up regulation in both AD and APP knockout mice could result from a loss of particular function of APP in AD, related to memory formation.

## References

[1] Bray SJ (2006) Notch signalling: a simple pathway becomes complex. Nat Rev Mol Cell Biol 7: 678-689. doi:10.1038/nrm2009. PubMed: 16921404. 16921404 10.1038/nrm2009

[2] Aydin D, Weyer SW, Müller UC (2012) Functions of the APP gene family in the nervous system: insights from mouse models. Exp Brain Res 217: 423-434. doi:10.1007/s00221-011-2861-2. PubMed: 21931985. 21931985 10.1007/s00221-011-2861-2

[3] Cao X, Sudhof TC (2001) A transcriptively active complex of APP with Fe65 and histone acetyltransferase Tip60. Science 293: 1436-1436. doi:10.1126/science.293.5534.1436. 10.1126/science.1058783 11441186

[4] von Rotz RC, Kohli BM, Bosset J, Meier M, Suzuki T et al. (2004) The APP intracellular domain forms nuclear multiprotein complexes and regulates the transcription of its own precursor. J Cell Sci 117: 4435-4448. doi:10.1242/jcs.01323. PubMed: 15331662. 15331662 10.1242/jcs.01323

[5] Zhang YW, Wang R, Liu Q, Zhang H, Liao FF et al. (2007) Presenilin/gamma-secretase-dependent processing of beta-amyloid precursor protein regulates EGF receptor expression. Proc Natl Acad Sci U S A 104: 10613-10618. doi:10.1073/pnas.0703903104. PubMed: 17556541. 17556541 10.1073/pnas.0703903104 PMC1888796

[6] Pardossi-Piquard R, Petit A, Kawarai T, Sunyach C, Alves da Costa C et al. (2005) Presenilin-dependent transcriptional control of the Abeta-degrading enzyme neprilysin by intracellular domains of betaAPP and APLP. Neuron 46: 541-554. doi:10.1016/j.neuron.2005.04.008. PubMed: 15944124. 15944124 10.1016/j.neuron.2005.04.008

[7] Müller T, Meyer HE, Egensperger R, Marcus K (2008) The amyloid precursor protein intracellular domain (AICD) as modulator of gene expression, apoptosis, and cytoskeletal dynamics-relevance for Alzheimer’s disease. Prog Neurobiol 85: 393-406. doi:10.1016/j.pneurobio.2008.05.002. PubMed: 18603345. 18603345 10.1016/j.pneurobio.2008.05.002

[8] Huysseune S, Kienlen-Campard P, Hébert S, Tasiaux B, Leroy K et al. (2009) Epigenetic control of aquaporin 1 expression by the amyloid precursor protein. FASEB J 23: 4158-4167. doi:10.1096/fj.09-140012. PubMed: 19687153. 19687153 10.1096/fj.09-140012

[9] Jones MW, Errington ML, French PJ, Fine A, Bliss TVP et al. (2001) A requirement for the immediate early gene Zif268 in the expression of late LTP and long-term memories. Nat Neurosci 4: 289-296. doi:10.1038/85138. PubMed: 11224546. 11224546 10.1038/85138

[10] Lee JL, Everitt BJ, Thomas KL (2004) Independent cellular processes for hippocampal memory consolidation and reconsolidation. Science 304: 839-843. doi:10.1126/science.1095760. PubMed: 15073322. 15073322 10.1126/science.1095760

[11] Lee JL (2008) Memory reconsolidation mediates the strengthening of memories by additional learning. Nat Neurosci 11: 1264-1266. doi:10.1038/nn.2205. PubMed: 18849987. 18849987 10.1038/nn.2205

[12] Maddox SA, Monsey MS, Schafe GE (2011) Early growth response gene 1 (Egr-1) is required for new and reactivated fear memories in the lateral amygdala. Learn Mem 18: 24-38. PubMed: 21177377. 21177377 10.1101/lm.1980211 PMC3023969

[13] Renbaum P, Beeri R, Gabai E, Amiel M, Gal M et al. (2003) Egr-1 upregulates the Alzheimer’s disease presenilin-2 gene in neuronal cells. Gene 318: 113-124. doi:10.1016/S0378-1119(03)00766-2. PubMed: 14585504. 14585504 10.1016/s0378-1119(03)00766-2

[14] Lu Y, Li T, Qureshi HY, Han D, Paudel HK (2011) Early growth response 1 (Egr-1) regulates phosphorylation of microtubule-associated protein tau in mammalian brain. J Biol Chem 286: 20569-20581. doi:10.1074/jbc.M111.220962. PubMed: 21489990. 21489990 10.1074/jbc.M111.220962 PMC3121503

[15] Vermeiren C, Najimi M, Maloteaux JM, Hermans E (2005) Molecular and functional characterisation of glutamate transporters in rat cortical astrocytes exposed to a defined combination of growth factors during in vitro differentiation. Neurochem Int 46: 137-147. doi:10.1016/j.neuint.2004.08.004. PubMed: 15627514. 15627514 10.1016/j.neuint.2004.08.004

[16] Morel M, Héraud C, Nicaise C, Suain V, Brion JP (2012) Levels of kinesin light chain and dynein intermediate chain are reduced in the frontal cortex in Alzheimer’s disease: implications for axoplasmic transport. Acta Neuropathol 123: 71-84. doi:10.1007/s00401-011-0901-4. PubMed: 22094641. 22094641 10.1007/s00401-011-0901-4

[17] Dovey HF, John V, Anderson JP, Chen LZ, de Saint Andrieu P et al. (2001) Functional gamma-secretase inhibitors reduce beta-amyloid peptide levels in brain. J Neurochem 76: 173-181. PubMed: 11145990. 11145990 10.1046/j.1471-4159.2001.00012.x

[18] Guan J-S, Haggarty SJ, Giacometti E, Dannenberg J-H, Joseph N et al. (2009) HDAC2 negatively regulates memory formation and synaptic plasticity. Nature 459: 55-U58. doi:10.1038/nature07925. PubMed: 19424149. 19424149 10.1038/nature07925 PMC3498958

[19] Sarantis K, Antoniou K, Matsokis N, Angelatou F (2012) Exposure to novel environment is characterized by an interaction of D1/NMDA receptors underlined by phosphorylation of the NMDA and AMPA receptor subunits and activation of ERK1/2 signaling, leading to epigenetic changes and gene expression in rat hippocampus. Neurochem Int 60: 55-67. doi:10.1016/j.neuint.2011.10.018. PubMed: 22080157. 22080157 10.1016/j.neuint.2011.10.018

[20] Gómez Ravetti M, Rosso OA, Berretta R, Moscato P (2010) Uncovering molecular biomarkers that correlate cognitive decline with the changes of hippocampus’ gene expression profiles in Alzheimer’s disease. PLOS ONE 5: e10153. doi:10.1371/journal.pone.0010153. PubMed: 20405009. 20405009 10.1371/journal.pone.0010153 PMC2854141

[21] MacGibbon GA, Lawlor PA, Walton M, Sirimanne E, Faull RL et al. (1997) Expression of Fos, Jun, and Krox family proteins in Alzheimer’s disease. Exp Neurol 147: 316-332. doi:10.1006/exnr.1997.6600. PubMed: 9344557. 9344557 10.1006/exnr.1997.6600

[22] Kim HS, Kim EM, Lee JP, Park CH, Kim S et al. (2003) C-terminal fragments of amyloid precursor protein exert neurotoxicity by inducing glycogen synthase kinase-3beta expression. FASEB J 17: 1951-1953. PubMed: 12923068. 12923068 10.1096/fj.03-0106fje

[23] Ryan KA, Pimplikar SW (2005) Activation of GSK-3 and phosphorylation of CRMP2 in transgenic mice expressing APP intracellular domain. J Cell Biol 171: 327-335. doi:10.1083/jcb.200505078. PubMed: 16230462. 16230462 10.1083/jcb.200505078 PMC2171208

[24] Belyaev ND, Nalivaeva NN, Makova NZ, Turner AJ (2009) Neprilysin gene expression requires binding of the amyloid precursor protein intracellular domain to its promoter: implications for Alzheimer disease. EMBO Rep 10: 94-100. doi:10.1038/embor.2008.222. PubMed: 19057576. 19057576 10.1038/embor.2008.222 PMC2613207

[25] Belyaev ND, Kellett KA, Beckett C, Makova NZ, Revett TJ et al. (2010) The transcriptionally active amyloid precursor protein (APP) intracellular domain is preferentially produced from the 695 isoform of APP in a {beta}-secretase-dependent pathway. J Biol Chem 285: 41443-41454. doi:10.1074/jbc.M110.141390. PubMed: 20961856. 20961856 10.1074/jbc.M110.141390 PMC3009870

[26] Goodger ZV, Rajendran L, Trutzel A, Kohli BM, Nitsch RM et al. (2009) Nuclear signaling by the APP intracellular domain occurs predominantly through the amyloidogenic processing pathway. J Cell Sci 122: 3703-3714. doi:10.1242/jcs.048090. PubMed: 19773363. 19773363 10.1242/jcs.048090

[27] Flammang B, Pardossi-Piquard R, Sevalle J, Debayle D, Dabert-Gay AS et al. (2012) Evidence that the amyloid-β protein precursor intracellular domain, AICD, derives from β-secretase-generated C-terminal fragment. J Alzheimers Dis 30: 145-153. PubMed: 22406447. 22406447 10.3233/JAD-2012-112186

[28] Beckett C, Nalivaeva NN, Belyaev ND, Turner AJ (2012) Nuclear signalling by membrane protein intracellular domains: the AICD enigma. Cell Signal 24: 402-409. doi:10.1016/j.cellsig.2011.10.007. PubMed: 22024280. 22024280 10.1016/j.cellsig.2011.10.007

[29] Pardossi-Piquard R, Checler F (2012) The physiology of the β-amyloid precursor protein intracellular domain AICD. J Neurochem 120 Suppl 1: 109-124. doi:10.1111/j.1471-4159.2011.07475.x. PubMed: 22122663. 22122663 10.1111/j.1471-4159.2011.07475.x

[30] Bauer C, Pardossi-Piquard R, Dunys J, Roy M, Checler F (2011) γ-Secretase-mediated regulation of neprilysin: influence of cell density and aging and modulation by imatinib. J Alzheimers Dis 27: 511-520. PubMed: 21841248. 21841248 10.3233/JAD-2011-110746

[31] Xu X, Zhou H, Boyer TG (2011) Mediator is a transducer of amyloid-precursor-protein-dependent nuclear signalling. EMBO Rep 12: 216-222. doi:10.1038/embor.2010.210. PubMed: 21293490. 21293490 10.1038/embor.2010.210 PMC3059912

[32] Hong Y, Beckett C, Belyaev ND, Turner AJ (2012) The impact of amyloid precursor protein signalling and histone deacetylase inhibition on neprilysin expression in human prostate cells. Int J Cancer 130: 775-786. doi:10.1002/ijc.26028. PubMed: 21365649. 21365649 10.1002/ijc.26028

[33] Peleg S, Sananbenesi F, Zovoilis A, Burkhardt S, Bahari-Javan S et al. (2010) Altered Histone Acetylation Is Associated with Age-Dependent Memory Impairment in Mice. Science 328: 753-756. doi:10.1126/science.1186088. PubMed: 20448184. 20448184 10.1126/science.1186088

[34] Li H, Wang B, Wang Z, Guo Q, Tabuchi K et al. (2010) Soluble amyloid precursor protein (APP) regulates transthyretin and Klotho gene expression without rescuing the essential function of APP. Proc Natl Acad Sci U S A 107: 17362-17367. doi:10.1073/pnas.1012568107. PubMed: 20855613. 20855613 10.1073/pnas.1012568107 PMC2951422

[35] Chasseigneaux S, Dinc L, Rose C, Chabret C, Coulpier F et al. (2011) Secreted amyloid precursor protein β and secreted amyloid precursor protein α induce axon outgrowth in vitro through Egr1 signaling pathway. PLOS ONE 6: e16301. doi:10.1371/journal.pone.0016301. PubMed: 21298006. 21298006 10.1371/journal.pone.0016301 PMC3029320

[36] Dickey CA, Loring JF, Montgomery J, Gordon MN, Eastman PS et al. (2003) Selectively reduced expression of synaptic plasticity-related genes in amyloid precursor protein + presenilin-1 transgenic mice. J Neurosci 23: 5219-5226. PubMed: 12832546. 12832546 10.1523/JNEUROSCI.23-12-05219.2003 PMC6741153

